# California State Veterinary Association

**Published:** 1891-03

**Authors:** Thomas Maclay

**Affiliations:** President


### SOCIETY PROCEEDINGS.

*California State Veterinary Medical Association.*—In February, 1888, Drs. Maclay, Fitzgerald, Bowhill, all members of the Royal College of Veterinary Surgeons, met in an office in San Francisco. A discussion of the merits of the veterinary profession and of the important issues relating to it, which needed guidance and regulation for the protection of the people, for the benefit of animals and for the profession itself, resulted in a resolution to form a State organization. A call was made to the Veterinary Profession in California through the Public Press. A few veterinary surgeons responded: an organization was affected. Dr. Thomas Bowhill was chosen President and temporary officers were appointed to carry on the business of the Association. At a subsequent meeting, 13th of March of the same year, a number of others joined and the constitution and by-laws were adopted. On the 28th of January, 1889, the association was incorporated under the laws of California, naming as directors for the first year, Dr. Thomas Maclay, of Petaluma, Chairman; Dr. Thomas Bowhill, of San Francisco, and Dr. A. M. McCallum, of Sacramento.

Under the incorporation a new set of By-Laws were adopted and as officers for the ensuing year Dr. Thomas Maclay was elected President; Dr. W. E. D. Morrison, Vice-President; Dr. A. M. McCallum, Secretary; and Dr. W. H. Woodruff, Treasurer. A Board of Examiners was also elected. At about this time the Directors had a bill presented to the California Legislature then in session at the capital of the state, entitled "An Act to Regulate the Practice of Veterinary Medicine and Surgery in the State of California." The bill passed the Senate and Assembly, but upon reconsideration in the lower house was beaten by four votes. After the defeat of the bill the association continued to hold its quarterly meetings and in December, A. D., 1889, the annual meeting was in Los Angeles, at which the officers named as above were re-elected for the ensuing year. In the meantime the Association continued to grow in strength and the benefit of organization began to show itself, and to-day there are very few graduates in the commonwealth of the Golden State who are not members of the Association.

*The Annual Meeting of the Association for 1890* was held on the 11th and 12th days of December, at the Capitol Hotel, Sacramento.

On the evening of the 11th, the meeting was called to order with President Thomas Maclay in the chair. The following named members being present: Drs. Morrison (Vice-President) and Witherspoon, of Los Angeles; Dr. Rowland, of Pasadena; Drs. Masoero, Egan, and Woodruff, of San Francisco; Dr. Orris, of Stockton; Dr. Spencer, of San José; Dr. Wadams, of Santa-Clara; Dr. Parent, of Hollister, and Dr. McCallum, of Sacramento.

Letters of regret for non-attendance were read from Drs. Blackington, Whittlesey, and Oliver, of Los Angeles; Dr. Pierce, of San Diego, and Dr. Burns, of San Francisco.

The minutes of the last quarterly meeting were read and approved. The annual reports of the Secretary, Treasurer, and Finance Committee were read, and on motion the same were adopted. The election of officers for the ensuing year resulted as follows: President, Thomas Maclay, of Petaluma; Vice:President, W. E. D. Morrison, of Los Angeles; Secretary, A. M. McCallum, Sacramento; Treasurer, W. H. Woodruff, San Francisco. Board of Directors: Dr. H. A. Spencer, Chairman, of San José; Dr. W. E. Wadams, of Santa Clara, and R. S. Whittlesey, of Los Angeles. Board of Examiners: Dr. Thomas Maclay, Chairman, of Petaluma; Dr. C. Masoero, of San Francisco; Dr. T. B. Rowland, of Pasadena; Dr. W. E. D. Morrison, of Los Angelos, and Dr. W. F. Egan, of San Francisco.

Dr. H. A. Spencer, of San Jose, read a paper on Emasculation of Horses. He regretted the numbers of traveling gelders "thoroughly ignorant of the parts they assail, or the pathology of the many untoward sequals," while acknowledging the expert proficiency which they may attain, he regrets that "This fact is more lamentable because these men not unfrequently make their operations a stepping-stone towards grappling with more serious matters, in the way of treatment of diseases and injuries." He pointed out the merits of the "Spencer hobbles." When ligation is used he prefers cotton to silk as the former rots and comes away with the slough. He denominated as "flankers," that class of horse, "where one testicle has not descended into the scrotum, but lies external to the abdominal wall and above Pouparts ligament and is held there by the pressure of the thigh," incontra-distinction to "ridglings" which he classes as those in which the testicle has not descended, and where "there is no canal."

He finds that the "yard-reared" colt requires much more attention after operating, than those raised and kept at pasture.

The paper called up a very lively discussion not only regarding the various methods of operating but also the several methods of casting colts and horses. The meeting then adjourned to meet the following day (the 12th) at 2 o'clock P. M.

On the morning of the 12th the members met at Dr. McCallum's Infirmary for the purpose of witnessing a practical illustration of Dr. Spencer's hobbles, which gave great satisfaction. Throwing horses with a single rope was also practically demonstrated by Dr. Rowland, of Pasadena. A "four-in-hand" took the members to the Rancho Del Paso, the property of J. B. Haggin, Esq., where lunch was served, the Superintendent acting as host. The different "corrals" were visited, the get of the great thoroughbreds were examined, Salvator, Hidalgo, Midlothian, Ben Ali, Joe Daniels, Miss Woodford, etc., etc.

At 2 o'clock P. M. the association was called to order. All the members present on the previous day were in attendance.

The secretary read a letter from Dr. Creely, the essayist of San Francisco, stating that he would be unable to attend the meeting.

Drs. Morrison, Rowland, Spencer, and Masoero, then described some important cases that had come under their notice since the last quarterly meeting of the association.

The subject matter of the bill to Regulate the Practice of Veterinary Medicine and Surgery in the State of California, was then taken up and discussed, preparatory to the presentation of the same to the Legislature now in session. After several amendments had been made to some of the sections of said bill, a resolution was introduced calling for the appointment —by the chair—of a committee of three, for the purpose of having said bill presented to the present Legislature, also, to urge the final passage of the same, whereupon, the president appointed Drs. Rowland, McCallum, and Egan, to act as such committee.

At the commencement of this meeting an application for membership was read from Dr. Davenfort, of Santa Rosa, the same was referred to the Board of Examiners, and was reported favorably. Dr. Davenfort was admitted a member of the association.

The meeting then adjourned, to meet in San Francisco in March. In the evening the annual dinner took place, the president occupied the chair. Plates were laid for twenty-five, members and visitors. The usual patriotic toasts were given and responded to.

THOMAS MACLAY, M. R. C. V. S. *President.*

